# Neurophysiological Effects of Meditation Based on Evoked and Event Related Potential Recordings

**DOI:** 10.1155/2015/406261

**Published:** 2015-06-07

**Authors:** Nilkamal Singh, Shirley Telles

**Affiliations:** Patanjali Research Foundation, Patanjali Yogpeeth, Haridwar, Uttarakhand 249405, India

## Abstract

Evoked potentials (EPs) are a relatively noninvasive method to assess the integrity of sensory pathways. As the neural generators for most of the components are relatively well worked out, EPs have been used to understand the changes occurring during meditation. Event-related potentials (ERPs) yield useful information about the response to tasks, usually assessing attention. A brief review of the literature yielded eleven studies on EPs and seventeen on ERPs from 1978 to 2014. The EP studies covered short, mid, and long latency EPs, using both auditory and visual modalities. ERP studies reported the effects of meditation on tasks such as the auditory oddball paradigm, the attentional blink task, mismatched negativity, and affective picture viewing among others. Both EP and ERPs were recorded in several meditations detailed in the review. Maximum changes occurred in mid latency (auditory) EPs suggesting that maximum changes occur in the corresponding neural generators in the thalamus, thalamic radiations, and primary auditory cortical areas. ERP studies showed meditation can increase attention and enhance efficiency of brain resource allocation with greater emotional control.

## 1. Introduction

Meditation has been described as a training in awareness which over long periods of time produces definite changes in perception, attention, and cognition. The neurophysiological correlates of meditation have been determined by electrophysiological recordings (from the 1960s to the present time) and more recently by neuroimaging studies (from the 1980s till the present time). Among electrophysiological variables sensory evoked potentials (EPs) provide a relatively noninvasive way of studying changes in specific sensory pathways during meditation [1]. It is believed that meditation alters cortical functioning and corticofugal controls which may significantly modify the processing of information at brainstem and thalamic levels [2–4]. Hence short, mid, and long latency EPs would be expected to help map changes from the brainstem up to the association or secondary cortical areas [5]. The present review was undertaken to determine which modalities and latencies of EPs were recorded in meditation and the conclusions derived.

EPs are evoked spontaneously with repetitive sensory stimulation and can provide information about brain resource allocation and the speed of stimulus processing, whereas event-related potentials (ERPs) are not spontaneous but elicited with cognitive task processing [6–8]. Hence ERPs can provide additional information regarding the discriminative ability of the brain and neurocognitive processing related to shifting attention. The present review also discusses studies which evaluated the effect of meditation on different modalities of ERPs.

## 2. Methods

### 2.1. Search Strategy for Meditation and Evoked Potential Studies

The database searched was PubMed using the search words “Meditation, Evoked Potentials.” Fifty-eight citations were obtained from PubMed. To be included in this review articles had to be written in English (8 articles were excluded as they were written in other languages). Articles were excluded from the review if (i) they reported event-related potentials (ERPs) rather than EPs (19 articles were excluded for this reason) and (ii) they did not deal directly with the subject of meditation (5 articles were excluded for this reason), (iii) the articles were not experimental studies but were review articles or descriptive (5 articles were excluded for this reason), and (iv) the study recorded variables other than EPs such as EEG, MRI, and spectroscopy studies (10 articles were excluded for this reason). Eleven articles reported evoked potential changes in different meditations and are reviewed here for their study design, method of meditation, and conclusions derived.

### 2.2. Search Strategy for Meditation and Event-Related Potential Studies

The databases searched were PubMed using the search words “Meditation, Event-Related Potentials.” Sixty citations were obtained from PubMed. To be included in this review articles had to be written in English (8 articles were excluded as they were written in other languages). Articles were excluded from the review if (i) they reported evoked potentials (EPs) rather than event-related potentials (ERPs) (15 articles were excluded for this reason) and (ii) they did not deal directly with the subject of meditation (3 articles were excluded for this reason), (iii) the articles were not experimental studies but were review articles or descriptive (5 articles were excluded for this reason), and (iv) the study recorded variables other than ERPs such as EEG, MRI, and spectroscopy studies (11 articles were excluded for this reason). Details of one study were not available. Seventeen articles reported event-related potential changes in different meditations and are reviewed here for their study design, method of meditation, and conclusions derived.

### 2.3. Method of Review

The whole papers were obtained and the details related to (i) stimulus modality for EPs or nature of the ERP, (ii) sweep width or latency of the EPs, (iii) type of meditation, (iv) study design, and (v) changes (if any) in EP or ERP components and the corresponding changes in the neural generators were noted.

## 3. Results

### 3.1. Details of the Evoked Potential Studies

Out of the eleven studies 10 used auditory stimuli while one used visual stimuli. With regard to sweep width out of the nine studies two reported short latency EPs, four mid latency EPs, two long latency EPs, and one long latency visual evoked potential (LLVEP) and there were also two combinations of (i) short latency and mid latency auditory EPs and (ii) short, mid, and long latency auditory EPs. Details about the components of EPs are given in Table 1.

The meditation techniques studied were all eyes closed practices; two were transcendental meditation (TM), two Qigong, five meditations on the Sanskrit syllable “OM,” one a moving meditation called cyclic meditation, and one Sahaja yoga meditation. Apart from the moving meditation the other eight techniques directed the thoughts in a fixed pattern for all practitioners towards either a single syllable or phrase or a set of thoughts. In the moving meditation as well, the sequence of thoughts was fixed and played on a CD [9].

Hence in all the studies the meditation techniques involved directing the attention in a specific way in all practitioners. The next point considered was the study design which included the controls used.

The range of experience in meditation was from naïve to twenty years. The sample size varied widely in the nine studies with a range between five and sixty practitioners and group average ± S.D. was 27.8 ± 18.4.

### 3.2. Details of the Event-Related Potential Studies

Out of the seventeen studies four used auditory oddball paradigm, three used the attentional blink task, two used mismatched negativity, two used affective picture viewing, one used global-to-local target task, one used the Stroop task, one used visual A-X continuous performance task, one used row/column speller, one used anticipatory and pain evoked ERPs, and one used discrimination of the imagery of the hand movement.

The meditation techniques used were Vipassana meditation in six studies, Sahaja yoga meditation, open monitoring meditation, Sudarshan Kriya yoga meditation, musical meditation, meditative mindfulness, cyclic meditation, mindfulness based cognitive therapy one each in seven studies, and two mixed traditions and 2 did not specify the meditation techniques used. All meditation techniques were performed with eyed closed.

### 3.3. Observations Common to Both EP and ERP Research in Meditation

In meditation research it is often difficult to find an appropriate control group which does not practice meditation but is otherwise comparable to the meditation group in other ways. This is because meditators often have other changes in their lifestyle such as abstaining from caffeine, nicotine, and other stimulating substances, different hours of rising and sleeping, and other differences. For this reason in some studies the meditators themselves are assessed under identical conditions on another day as a control, which may be described as the self-as-control [10].

This design was followed in four of the EP studies which are presented in Table 2 [5, 11–13]. There were two single group studies [1, 14]. These were the earliest studies on evoked potentials and meditation. The absence of a comparison or control state or group is an obvious disadvantage, as the effect of time during a session can be expected to influence the evoked potential components and the meditation effect would not be separated from this. In five other papers [15–19] the approach taken was to have separate groups of participants. In only one trial [17] the participants were randomized as three groups. The advantage of the separate groups design over the self-as-control is that nonmeditators would not get inadvertently into meditation which is a disadvantage of the self-as-control design, where experienced meditators may get into the meditative state automatically, even during a control session.

Out of the seventeen ERP studies, 12 studies had a two-group design, four studies had a self-as-control design, and one had mixed design in which the study was conducted in two phases, phase 1 was a cross sectional study and phase 2 was a longitudinal study. This is described in Table 3. The disadvantages and advantages are comparable to those described above for EP studies. With ERP studies there is an additional point; since the participants are given a task it is usual to record before and after meditation, not during meditation, as doing the task would interfere with meditation.

### 3.4. Results of EPs

#### 3.4.1. Auditory Evoked Potentials

Discussing the short latency or brainstem auditory evoked potentials first, there were three studies. In the earliest study [14] the peak latency of wave V which corresponds to the inferior colliculus varied with the intensity of the stimuli and hence could not be considered as an effect of the transcendental meditation (TM) practiced. In the second study which reported short latency auditory evoked potentials in Qigong meditators [15] there was a significant increase in the peak amplitudes of waves I to V of short latency auditory evoked potentials. An increase in amplitude is suggestive of recruitment of increased neurons in the underlying neural generators [20]. This suggested that Qigong meditation activates areas in the brainstem. There was no change in short latency auditory evoked potentials in patients with epilepsy who practiced Sahaja yoga possibly because they were novices to meditation [17]. In the other study which reported changes in short latency auditory evoked potentials the peak latency of wave V increased during random thinking, focusing and meditative focusing, but not during meditation [11]. An increase in peak latency signifies delayed transmission through the respective neural generators [5], in this case located in the brain stem. Hence it would appear as if random thinking, focusing, and meditative focusing all delay auditory information transmission at the brainstem level but this does not happen during meditation on “OM.”

The mid latency auditory evoked potentials correspond to neural generators in the thalamus, primary auditory cortex, and Haschl's gyrus [2–4]. With Qigong meditation the amplitudes of Na and Pa components decreased during meditation [15]. The Pa wave peak amplitude also decreased during meditation on “OM” in sixty practitioners [12] but there was an increase in peak amplitude of Na wave during meditation on “OM” in a separate study [18]. The Na wave is believed to be due to activity at the mesencephalic and diencephalic level [21] and the Pa wave corresponds to activity at the superior temporal gyrus [22]. A third component of the mid latency auditory evoked potentials is the Nb wave. This wave appears relatively localized in the dorso-posterior-medial area of Heschl's gyrus, that is, the primary auditory cortex [23]. The Nb wave peak amplitude increased and peak latencies of Pa and Nb waves decreased after the moving meditation called cyclic meditation (CM), which incorporates movement [5]. The peak latency of Nb wave reduced during meditation on “OM” as well [19]. Hence the moving meditation altered the auditory pathway at the level of superior temporal gyrus and Heschl's gyrus with a delay in auditory transmission despite an increase in number of neurons recruited. During meditation on “OM” the delay in auditory transmission was found only in the dorso-posterior-medial area of Heschl's gyrus.

Long latency auditory evoked potentials showed no significant change in an early study on transcendental meditation practitioners [1]. The P2 component showed a decrease in peak amplitude during Qigong meditation and a decrease in peak latency during meditation on “OM.” The P2 wave partly reflects auditory output of the mesencephalic activation system [24, 25]. From MEG and EEG data based on depth electrodes in patients, the neural generators for the P2 component were localized in the planum temporale as well as the auditory association complex (Brodmann area 22) [26–29]. Also it is speculated that the P2 component may receive contributions from cortical areas in the depth of the Sylvian fissure. Hence this area, which is associated with complex auditory functions, changes during both Qigong and OM meditations.

#### 3.4.2. Summary of Changes in Auditory Evoked Potentials

Out of the four studies which evaluated short latency auditory evoked potentials changes occurred in brainstem evoked potentials only in Qigong [15], not in transcendental meditation [14], in Sahaja yoga [17], or in “OM” meditation [11]. With respect to mid latency auditory evoked potentials assessed in four meditations changes were seen in all four meditations [5, 12, 15, 17–19], that is, Qigong, Sahaja yoga, meditation on “OM,” and cyclic meditation. This suggests that meditation modifies neural generators at the level of specific thalamic nuclei, thalamic radiation, and primary sensory cortices irrespective of the meditation techniques. Long latency AEPs changed in two out of the three meditations in which they were recorded. Hence there were changes during Qigong and “OM” meditation but not during TM.

#### 3.4.3. Visual Evoked Potentials

Visual evoked potentials were recorded in a single study on Qigong meditators [16]. There was a significant increase in peak-to-peak amplitude of N80-P115-N150 and N150-P200-N280 waves during Qigong meditation. The authors suggest that N80-P115-N150-P200 recorded may have corresponded to N70-P100-N130-P170 reported by Vaughan in 1996 [30]. The first positive component is believed to be generated within thalamocortical radiations; the subsequent negative component is generated in lamina IV cb. The next positive component reflects inhibitory activity within this lamina and the later positive component reflects extra striate cortex activity. This suggests that Qigong meditation increases the activity in the visual pathway from thalamocortical radiations up to the extra striate cortex with all the relay centers in between being included.

### 3.5. Results of ERPs

Healthy experienced Sahaja yoga meditators and nonmeditators were assessed in a two-group study using an affective picture viewing task [31]. In this comparison between long-term Sahaja yoga meditators and nonmeditators, mid latency ERPs were attenuated for both positive and negative pictures and a stronger ERP negativity between 200 and 300 ms was found in meditators regardless of picture valence.

There were two separate studies on healthy experienced Vipassana practitioners using an auditory oddball task [32, 33]. In both the studies the groups were assessed in two separate conditions (“the self as control” design), that is, Vipassana meditation and random thinking. In one of the studies Vipassana practitioners showed greater P3b amplitudes to the target tone after meditation than they did both before meditation and after the nonmeditation session [32]. The other study reported changes in multiple components in response to the standard and target stimuli; the meditation-induced reduction in P3a amplitude had a positive correlation with the quality and experience of meditation [33].

In another study healthy Vipassana meditators were given a three-stimulus auditory oddball task [34]. The group was assessed in two separate conditions, that is, Vipassana meditation and instructed mind wandering. Meditation compared to the control condition had decreased evoked delta power to distracter stimuli concomitantly with a greater event-related reduction of late (500–900 ms) alpha-1 (8–10 Hz) activity, suggestive of a modification of attentional engagement to distracters. Additionally, standard stimuli were associated with increased early event-related alpha phase synchrony (intertrial coherence) and evoked theta phase synchrony. Finally, during meditation, there was a greater differential early-evoked gamma power to the different stimuli. Correlation analysis indicated that this effect from a meditation state-related increase in early distracter-evoked gamma power and phase synchrony was specific to longer-term expert practitioners.

Two separate studies compared healthy Vipassana meditators and nonmeditators using the attentional blink task [35, 36]. In one study three months of intensive mental training resulted in a smaller attentional blink and reduced brain-resource allocation to the first target, as reflected by a smaller T1-elicited P3b, a brain-potential index of resource allocation [35]. This efficiency in brain resource allocation might explain the findings of the other study which reported changes, especially in theta phase locking in conscious target perception [36].

Healthy practitioners of Vipassana and loving kindness meditation and nonmeditators were assessed in an attention blink task and an attention auditory task [37]. Hence this two-group study compared practitioners of Vipassana and loving kindness meditation with nonmeditators. Three months of intensive meditation training reduced variability in attentional processing of target tones and reduced reaction time variability. Those individuals with greatest increase in neural response consistency had largest decrease in behavioral response variability. Reduced variability in neural processing was observed regardless of whether the deviant tone was attended to or unattended. Meditation can significantly affect attention and brain function.

Performance in a Global-to-Local target task was compared between healthy open monitoring meditators and nonmeditators [38]. The study was conducted in two phases. Phase 1 was a cross sectional study. Phase 2 was a longitudinal study. Open monitoring meditation practitioners showed an enhanced processing of target level information, in contrast to the control group, which showed a local target selection effect only in the P1 and a global target selection effect in the P3 component; meditators showed effects of local information processing in the two other components (other than P3) and of global processing for two other components. Thus, meditators seem to display enhanced depth of processing. In the longitudinal experiment, meditation modulated attention after a 4-day meditation retreat.

Two studies reported the effect of meditation on mismatch negativity (MMN) [39, 40] Healthy practitioners of Sudarshan Kriya yoga and nonmeditators were compared using the mismatch negativity (MMN) paradigm [39]. In this two-group, comparative study Sudarshan Kriya yoga meditators were found to have larger MMN amplitudes than nonmeditators. Meditators also exhibited significantly increased MMN amplitudes immediately after meditation suggesting transient state changes owing to meditation. The MMN is related to neuropsychological functioning, particularly to executive functions [41]. Another study on healthy comparing children with training in musical meditation and nonmeditators using auditory mismatch negativity (MMN) and P300 [40] reported similar results.

Apart from the mismatch negativity (MMN) paradigm P300 can also be a useful indicator for neuropsychological functioning such as brain resource allocation for attentional processing. Two separate studies on two different types of meditation supported the findings mention in the above paragraph [42, 43]. Healthy practitioners of meditative mindfulness and a control group were assessed using a row/column speller task [42]. In this two-group study, the meditative mindfulness induction (MMI) and non-MMI control group were compared. MMI subjects were significantly more accurate than control subjects and they produced significantly larger P300 amplitudes than control subjects at the vertex and parietooccipital region. In a separate study, practitioners of a moving meditation called cyclic meditation were assessed on two separate days, practicing cyclic meditation or an equal duration of supine rest for comparison [43]. On both days they were given an auditory oddball task with two tones (standard and target). There was a reduction in the peak latency of P300 after cyclic meditation at the frontal region, vertex, and parietal region. Also the P300 peak amplitudes after CM were higher at the same sites.

In addition to the studies on healthy participants the effect of meditation was evaluated on patients with bipolar disorder which is characterized by a number of attentional abnormalities. Patients with bipolar disorder and normal healthy participants were assessed with a visual A-X continuous performance task [44]. In this two-group study, mindfulness based cognitive therapy in bipolar disorder improved attentional readiness and attenuated activation of nonrelevant information processing during attentional processes.

Four separate studies compared meditators and nonmeditators using different tasks for ERPs [45–48]. In one of the studies healthy meditators of various traditions and nonmeditators performed the Stroop task [45]. Meditators showed greater executive control (i.e., fewer errors), a higher error related negativity, and emotional acceptance than controls. Another study used emotionally loaded stimuli (i.e., the International Affective Picture System) [46]. The result showed different emotional processing in meditation practitioners. At high levels of processing meditators are less affected by stimuli with an adverse emotional load, while processing of positive stimuli remains unaltered. In another study healthy meditators from different traditions and nonmeditators were assessed using anticipatory and pain-evoked ERPs [47]. Meditation was found to reduce the anticipation and negative appraisal of pain. Practitioners of imaginative meditators and nonmeditators were assessed during hand movement(s) and the idle thinking state [48]. Imagining moving the hand is associated with event-related desynchronization of the beta EEG. This occurred more often and predictably in meditators compared to controls.

#### 3.5.1. Summary of Event-Related Potential Studies

The results of the study on Sahaja yoga practitioners showed meditation can enhance frontal top-down control over fast automatic salience detection, based on amygdala functions following long-term meditation [31]. Studies on Vipassana meditators showed increased attentional engagement after meditation, enhanced perceptual clarity, decreased automated reactivity, increased efficiency in distribution of limited brain resources, and switching attention [32–37]. Similar findings were reported in studies done on open monitoring meditation, Sudarshan Kriya yoga, musical meditation, and meditative mindfulness and cyclic meditation [38–43]. Meditators were also reported to have greater emotional acceptance, were less affected by stimuli with an adverse emotional load, and reduced the anticipation and negative appraisal of pain [45–47]. Hence meditation can induce a mental state which is characterized by efficient brain resource allocation with greater emotional control.

## 4. Limitations of EP and ERP Studies Reviewed

One of the main limitations of the studies reviewed is in the study design. Out of the 11 EP studies there was just one [17] which randomized the participants to the three experimental conditions. The other multiple-group studies [16, 18–21] did not use randomization. Three other early studies [1, 14, 15] had a single group which is an obvious disadvantage. The remaining 4 studies assessed the meditators as their own control on a separate day or days. In one out of four studies the assignment to meditation or the control intervention was random [5], whereas in the other 3 studies [11–13] the sequence was fixed and hence the effect of one intervention on another could not be ruled out. Also a disadvantage of the self-as-control design is that meditators with long experience in meditation may get into a meditative state even during the nonmeditation control sessions. Apart from the disadvantages in study design another disadvantage especially in the early studies [1, 14–16] was small sample sizes (range between 5 and 15). This is not seen in more recent studies [12, 13] (*n* = 60 and *n* = 48, resp.). On the whole evoked potentials offer poor spatial and temporal resolution compared to fMRI and MEG. Also the localization of changes is restricted to the sensory pathway activated by the specific stimulus. Despite these limitations evoked potentials continue to be useful to studies on practices like meditation as they are far less distracting and do not involve a drastic change in posture as is required for fMRI.

In the ERP studies reviewed the number of multiple-group studies (12 studies) was higher than the EP studies reviewed in the present study. However the evidence of the ERP studies is also with similar limitations as none of the ERP studies used randomization as a method of allocating the participants to different groups.

## 5. Limitations of the Present Study

The present study has several limitations: (i) the present study did not review studies on meditation and EEG, (ii) the studies on meditation and EPs were not homogenous, (iii) the studies reviewed in the present study were not categorized according to the Jadad scale, and (iv) the studies included in the present study were searched from only one database, that is, PubMed.

## 6. Directions for Future Research Suggested by This Study

The present study has shown that EPs are useful in localizing changes in meditation to areas such as the brainstem, thalamus, thalamocortical radiations, primary sensory cortices, and association cortical areas and ERPs can provide useful information about neurocognitive processing of attention and brain resource allocation. Most of the studies are limited by small sample sizes, lack of proper controls, no objective way of assessing the quality of meditation, and a wide range of variation in the practitioners sampled. This can be corrected in future studies. Also studies can be specifically designed to verify whether the findings of EP studies which suggest that changes occur in the thalamus, thalamocortical connections, and primary relays and enhancement in attention and increased efficiency of brain resource allocation as suggested by ERP studies are indeed correct. To verify these results more rigorous studies with a better design, larger sample size and studies of EPs and ERPs in combination with neuroimaging during meditation are recommended.

## Figures and Tables

**Table 1:** Neural generators of evoked potential components.

S. number		Name of the components	Latencies (msec)	Neural generators
1	Short latency auditory evoked potential	Wave I	1.9	Auditory portion of the eighth cranial nerve
		Wave II	3.6	Near or at the cochlear nucleus. A portion from the eighth nerve fibers around the cochlear nucleus
		Wave III	4.2	The lower pons through the superior olive and trapezoid body
		Wave IV	5.2	The upper pons or lower midbrain, in the lateral lemniscus and the inferior colliculus; a contralateral brainstem generator for wave V is suggested
		Wave V	5.8	
		Na	14–19	Medial geniculate body
		Pa	25–32	Superior temporal gyrus

2	Mid latency auditory evoked potential	Nb	35–65	Dorso-posterior-medial part of Heschl's gyrus that is the primary auditory cortex
		N1	40–60 ms	Secondary auditory cortex in the lateral Heschl's gyrus
		P1	80–115 ms	Bilateral parts of the auditory superior cortex

	Long latency auditory evoked potential	N2	140–180 ms	Mesencephalic-reticular activating system (RAS)
		P2	220–280 ms	Anterior cingulate cortex

**Table 2:** Details of the evoked potential studies.

S. number	Reference	Modality auditory/visual/somatosensory and latency	Type of meditation	Meditation experience, duration	Components altered and brain area	Sample and design
1	Electroencephalogr Clin Neurophysiol. 1978, 45 (5): 671–673 [1]	Auditory Long latency	Transcendental meditation	18 months to 6 years	No significant change	Single group

2	Intern J Neuroscience. 1980, 10 (2-3): 165–170 [14]	Auditory Short latency	Transcendental meditation	6 to 9 years	Wave V latency increased in moderate intensity stimuli and wave V latency decreased in high intensity stimuli	Single group

3	Am J Chin Med. 1990, 18 (3-4): 95–103 [15]	Auditory Short latency, middle latency, and long latency	Qigong meditation which involves the initial “concentrating,” a subsequent “circulating,” and finally the “dispersion” of Qi	1 to 20 years	There was a significant increase in amplitude in wave I-V of BAER, Na and Pa wave of MLR decreased, and P2 wave of LLAER also decreased during meditation	3 groups: BAER, MLAER, and LLAER

4	Am J Chin Med. 1993, 21 (3-4): 243–249 [16]	Visual Cortical evoked potentials	Qigong meditation in which the practitioner concentrates on the “Dantian”	2.3 years for the experienced group, 1.9 months for the learning group	Peak-to-peak amplitude of N80-P115-N150 and N150-P200-N280 increased in the experienced group	3 groups: practitioner group learning group, and control group

5	Int J Neurosci. 1994; 76 (1-2): 87–93 [18]	Auditory Middle latency evoked potentials	“OM” meditation in which the participants meditated with effortless absorption in the single-thought state of the object of meditation, that is, “OM.”	10 years	Experienced meditators had significant increase in peak amplitude of Na wave during meditation and significant reduction in Na wave peak amplitude during control session	Two-group study (experienced meditators and nonexperienced)

6	Indian J Med Res. 1993; 98: 237–9 [19]	Auditory Middle latency evoked potentials	“OM" meditation in which the participants meditated with effortless absorption in the single-thought state of the object of meditation, that is, “OM.”	5–20 years	Experienced meditators had a significant reduction in the peak latency of the Nb wave	Two-group study (experienced meditators and nonexperienced)

7	Appl Psychophysiol Biofeedback. 2000, 25 (1): 1–12 [17]	Auditory BAEP MLAEP	Sahaja yoga in which the participants make certain mental assertions by placing the hand on different parts of the body.	Not experienced *N* = 10 (Sahaja yoga group), *N* = 10 (mimicking exercise group), and *N* = 12 (control group)	Significant increase in Na-Pa amplitude of MLR following meditation practice	Randomized controlled study

8	Clin EEG Neurosci. 2009, 40 (3): 190–195 [5]	Auditory Mid latency	Cyclic meditation in which a series of *asanas* (postures interspersed with relaxation techniques) are practiced with awareness.	6–48 months	After cyclic meditation there was a significant increase in the peak latency of the Pa wave and of the Nb wave; peak amplitude of the Nb wave also increased	Self as control design

9	Int J Yoga. 2010 3 (2): 37–41 [11]	Auditory Brainstem auditory-evoked potentials (BAEPs)	“OM” meditation in which the participants meditated with effortless absorption in the single-thought state of the object of meditation, that is, “OM.”	6 months	Wave V peak latency significantly increased in cancalata, ekagrata, and dharana, but no change occurred during the dhyana session	Self as control

10	Clin EEG Neurosci. 2012, 43 (2): 154–60 [12]	Auditory Mid latency	“OM” meditation participants were instructed to keep their eyes closed and dwell on thoughts of OM, without any effort, particularly on the subtle (rather than physical) attributes and connotations of the syllable.	6–60 months	Significant increase in the peak latencies of Na and Pa waves during meditation and the peak amplitude of Pa wave was significantly decreased during meditation	Self as control design

11	Clin EEG Neurosci. 2014, pii: 1550059414544737 [13]	Auditory Long Latency	“OM” meditation in which participants were instructed to keep their eyes closed and dwell on thoughts of OM, without any effort, particularly on the subtle (rather than physical) attributes and connotations of the syllable.	6–60 months	Decrease in the peak latency of the P2 wave during and after meditation	Self as control design

**Table 3:** Details of event related studies.

S. number	Citation	Participants	Nature of the ERP task	Design	Intervention	Findings
1	Neuroscience. 2014, 281C: 195–201 [31]	Healthy experienced meditators and nonmeditators	Affective picture viewing	Two-group study	No intervention was given but this study compared between long-term experienced Sahaja yoga meditators and nonmeditators	Mid latency (140–400 ms) ERPs were attenuated for both positive and negative pictures and a stronger ERP negativity in the time window 200–300 ms was found in meditators regardless of picture valence. We assume that long-term meditation practice enhances frontal top-down control over fast automatic salience detection, based on amygdala functions.

2	Int J Psychophysiol. 2013, 90 (2): 207–214 [32]	Healthy experienced meditators aged 20–61 years	Auditory oddball task with two tones (standard and target)	One group was assessed in two separate conditions (self as control)	Vipassana meditation and random thinking	The Vipassana experts showed greater P3b amplitudes to the target tone after meditation than they did both before meditation and after the no-meditation session. These results suggest that expert Vipassana meditators showed increased attentional engagement after meditation.

3	Soc Cogn Affect Neurosci. 2013, 8 (1): 100–111 [34]	Healthy Vipassana meditators Exp. = 2.5–40 years	Three-stimulus auditory oddball task	One group was assessed in two separate conditions (self as control)	Vipassana meditation and instructed mind wandering	Meditation compared to control condition had decreased evoked delta (2–4 Hz) power to distracter stimuli concomitantly with a greater event-related reduction of late (500–900 ms) alpha-1 (8–10 Hz) activity, which indexed altered dynamics of attentional engagement to distracters. Additionally, standard stimuli were associated with increased early event-related alpha phase synchrony (intertrial coherence) and evoked theta (4–8 Hz) phase synchrony, suggesting enhanced processing of the habituated standard background stimuli. Finally, during meditation, there was a greater differential early-evoked gamma power to the different stimulus classes. Correlation analysis indicated that this effect stemmed from a meditation state-related increase in early distracter-evoked gamma power and phase synchrony specific to longer-term expert practitioners. The findings suggest that Vipassana meditation evokes a brain state of enhanced perceptual clarity and decreased automated reactivity.

4	Front Hum Neurosci. 2012, 6: 133 [38]	Healthy meditators and nonmeditators	Global-to-local target task	Study conducted in two phases Phase 1: cross sectional study Phase 2: longitudinal study	Open monitoring meditation	Meditators showed an enhanced processing of target level information. In contrast with control group, which showed a local target selection effect only in the P1 and a global target selection effect in the P3 component, meditators showed effects of local information processing in the P1, N2, and P3 and of global processing for the N1, N2, and P3. Thus, meditators seem to display enhanced depth of processing. In the longitudinal experiment, meditation modulates attention already after a 4-day meditation retreat. Together, these results suggest that practicing meditation enhances the speed with which attention can be allocated and relocated, thus increasing the depth of information processing and reducing response latency

5	Soc Cogn Affect Neurosci. 2013, 8 (1): 85–92 [45]	Healthy meditators and nonmeditators	Stroop task	Two-group study	Comparison between meditators and nonmeditators, meditators are from various traditions	Meditators showed greater executive control (i.e., fewer errors), a higher error related negativity (ERN), and more emotional acceptance than controls.

6	BMC Psychiatry. 2012, 12: 15 [44]	Patients with bipolar disorder and normal healthy participants	A visual A-X continuous performance task	Two-group study	Mindfulness based cognitive therapy (MBCT)	MBCT in bipolar disorder improved attentional readiness and attenuated activation of nonrelevant information processing during attentional processes

7	J Neural Eng. 2011, 8 (2): 025019 [42]	Healthy individuals	Row/column speller task	Two-group study	Meditative Mindfulness Induction (MMI) and non-MMI control group	MMI subjects were significantly more accurate than control subjects and they produced significantly larger P300 amplitudes than control subjects at Cz and PO7

8	Neurosci Res. 2011, 71 (1): 44–48 [46]	Healthy meditators and nonmeditators	Emotional load of stimuli (IAPS pictures)	Two-group study	No intervention was given but this study compared with experienced meditators and nonmeditators	The result showed different emotional processing in meditation practitioners: at high levels of processing meditators are less affected by stimuli with adverse emotional load, while processing of positive stimuli remains unaltered

9	Pain. 2010, 150 (3): 428–438 [47]	Healthy meditators and nonmeditators	Anticipatory and pain-evoked ERPs	Two-group study	No intervention was given but this study compared with experienced meditators and nonmeditators; meditators were from different traditions	Meditation reduces the anticipation and negative appraisal of pain

10	J Neurosci. 2009, 29 (42): 13418–13427 [37]	Healthy meditators and nonmeditators	Attention blink task and attention auditory task	Two-group study	Vipassana and loving kindness meditation	Three months of intensive meditation training reduced variability in attentional processing of target tones and reduced reaction time variability. Those individuals with greatest increase in neural response consistency had largest decrease in behavioral response variability. Reduced variability in neural processing was observed regardless of whether the deviant tone was attended or unattended. *n* significantly affect attention and brain function.

11	Conf Proc IEEE Eng Med Biol Soc. 2008, 2008: 662–665. [48]	Meditators and nonmeditators	Discrimination of the imaginative hand movement and the idle state	Two-group study	Type of meditation not specified	The meditation practice can improve the classification accuracy of EEG patterns. The average classification accuracy was 88.73% in the meditation group, while it was 70.28% in the control group. An accuracy as high as 98.0% was achieved in the meditation group.

12	Int J Psychophysiol. 2009, 72 (1): 51–60. [33]	Healthy experienced meditators	Auditory oddball task with two tones (standard and target)	One group was assessed in two separate conditions (self as control)	Vipassana meditation and random thinking	During meditation N1 amplitude from the distracter was reduced frontally; P2 amplitudes from both the distracter and oddball stimuli were somewhat reduced; P3a amplitude from the distracter was reduced. The meditation-induced reduction in P3a amplitude had a positive correlation with the quality and experience of meditation

13	J Cogn Neurosci. 2009, 21 (8): 1536–1549. [36]	Healthy meditators and nonmeditators	The attentional blink task	Two-group study	Vipassana Meditation	Theta phase locking in conscious target perception and suggest that after mental training the cognitive system is more rapidly available to process new target information. Mental training was not associated with changes in the amplitude of T2-induced responses or oscillatory activity before task onset

14	Neuroreport. 2007, 18 (16): 1709–1712. [39]	Healthy meditators and nonmeditators	The mismatch negativity (MMN) paradigm	Two-group study	Sudarshan kriya yoga meditation	Meditators were found to have larger MMN amplitudes than nonmeditators. The meditators also exhibited significantly increased MMN amplitudes immediately after meditation suggesting transient state changes owing to meditation.

15	PLoS Biol. 2007, 5 (6): e138. [35]	Healthy meditators and nonmeditators	The attentional blink task	Two-group study	Vipassana	Three months of intensive mental training resulted in a smaller attentional blink and reduced brain-resource allocation to the first target, as reflected by a smaller T1-elicited P3b, a brain-potential index of resource allocation. Those individuals that showed the largest decrease in brain-resource allocation to T1 generally showed the greatest reduction in attentional blink size. These observations provide novel support for the view that the ability to accurately identify T2 depends upon the efficient deployment of resources to T1. The results also demonstrate that mental training can result in increased control over the distribution of limited brain resources.

16	Int J Neurosci. 2006, 116 (12): 1419–1430. [43]	Healthy individuals	Auditory oddball task with two tones (standard and target)	One group was assessed in two separate conditions (self as control)	Cyclic meditation	There was reduction in the peak latencies of P300 after cyclic meditation at Fz, Cz, and Pz compared to the “pre” values. The P300 peak amplitudes after CM were higher at Fz, Cz, and Pz sites compared to the “pre” values.

17	Chin Med Sci J. 1999, 4 (2): 75–79. [40]	Healthy meditators and nonmeditators	The auditory mismatch negativity (MMN) and P300	Two-group study	Musical meditation	MMN amplitudes in the trained children were larger than those in the control group. In addition, the MMN amplitudes were identical in attend and ignore conditions for both groups.
